# Preparation of Reactive Indicator Papers Based on Silver-Containing Nanocomposites for the Analysis of Chloride Ions

**DOI:** 10.3390/mi14091682

**Published:** 2023-08-28

**Authors:** Marina O. Gorbunova, Igor E. Uflyand, Vladimir A. Zhinzhilo, Anastasiya O. Zarubina, Tatiana S. Kolesnikova, Maxim G. Spirin, Gulzhian I. Dzhardimalieva

**Affiliations:** 1 Rostov State Medical University of the Ministry of Healthcare of Russian Federation, 344022 Rostov-on-Don, Russia; mg700@mail.ru; 2 Department of Chemistry, Southern Federal University, 344090 Rostov-on-Don, Russia; i06993@yandex.ru (V.A.Z.); karginova@sfedu.ru (A.O.Z.); shkip90@yandex.ru (T.S.K.); 3 Federal Research Center of Problems of Chemical Physics and Medicinal Chemistry, Russian Academy of Sciences; 142432 Chernogolovka, Russia; max2004@icp.ac.ru (M.G.S.); dzhardim@icp.ac.ru (G.I.D.); 4 Moscow Aviation Institute, National Research University, 125993 Moscow, Russia

**Keywords:** nanocomposites, metal-containing monomers, reactive indicator paper, chloride ions, analysis

## Abstract

In recent decades, metal-containing nanocomposites have attracted considerable attention from researchers. In this work, for the first time, a detailed analysis of the preparation of reactive indicator papers (RIPs) based on silver-containing nanocomposites derived from silver fumarate was carried out. Thermolysis products are silver-containing nanocomposites containing silver nanoparticles uniformly distributed in a stabilizing carbon matrix. The study of the optical properties of silver-containing nanocomposites made it possible to outline the prospects for their application in chemical analysis. RIPs were made by impregnating a cellulose carrier with synthesized silver fumarate-derived nanocomposites, which change their color when interacting with chlorine vapor. This made it possible to propose a method for the determination of chloride ions with preliminary oxidation to molecular chlorine, which is then separated from the solution by gas extraction. The subsequent detection of the active zone of RIPs using colorimetry makes it possible to identify mathematical dependences of color coordinates on the concentration of chloride ions. The red (R) color coordinate in the RGB (red-green-blue) system was chosen as the most sensitive and promising analytical signal. Calibration plots of exponential and linear form and their equations are presented. The limit of detection is 0.036 mg/L, the limits of quantification are 0.15–2.4 mg/L, and the time of a single determination is 25 min. The prospects of the developed technique have been successfully shown in the example of the analysis of the natural waters of the Don River, pharmaceuticals, and food products.

## 1. Introduction

Chloride ions are among the most abundant anions in nature and can be found in many objects. An excessive concentration of chlorides significantly impairs the taste of drinking water and can lead to the impossibility of using water for technical and agricultural purposes. Therefore, the content of chlorine ions, which is an important indicator of the quality of natural and drinking water, must be controlled. The development of new methods for the determination of chlorides is also relevant for several other areas, such as medicine and the food industry [1,2,3,4,5,6].

Among the most commonly used methods for determining chlorides, an important place is occupied by argentometry or spectrophotometry with mercury salts. These methods are simple, but they are characterized by a significant drawback associated with the use of many expensive reagents that form toxic waste [6]. Recently, various analytical methods for the detection of chlorides have been proposed. Thus, methods for determining chloride ions such as electrochemical [5,7,8,9], IR [1], optical emission spectroscopic [2], ion chromatographic [3,4], mass spectrometric [10], fluorometric [11], and spectrophotometric [5] methods are of considerable interest. It should be noted that a significant number of publications on the determination of chloride ions that have appeared in recent years indicate the relevance of this problem.

One of the promising areas of modern analytical chemistry is the development of test methods for on-line out-of-lab analysis using solid-phase analytical reagents. The most commonly used indicator forms are tablets, thin films, and reagents deposited on cellulose carriers, such as paper test strips [12,13,14]. The interaction of the analyte with the reagent manifests itself in a change in color due to the formation of a colored compound [15,16,17] or bleaching of the dye [18,19,20], and also leads to a change in the spectral properties of nanoparticles [21,22,23,24]. In other words, solid-phase analytical reagents represent a specific class of chemosensors. The study of the dependence of the change in the properties of reagents on the concentration of the analyte makes it possible to use them both in qualitative and quantitative analysis.

At present, metal nanoparticles (in particular, gold and silver) are widely used in analytical chemistry as components of chemosensors. They are used in various forms, such as colloidal solutions of nanoparticles, solid nanocomposites, etc. [25,26,27,28,29,30,31,32,33]. The operation of such sensors is based on the phenomenon of surface plasmon resonance. Among the undoubted advantages of such chemosensors, one should note the ease of fixing the analytical response, high sensitivity, and the possibility of fine tuning the optical and analytical characteristics. At the same time, they have several disadvantages, in particular, the aggregative instability of nanoparticles, which can be caused by a high ionic strength or the presence of substances that are specifically adsorbed on nanoparticles and destabilize them [34,35,36,37]. One of the widely used approaches to solving this problem is the complex modification of nanoparticles but this leads to a significant increase in the cost of analysis. A more optimal solution is the spatial separation of the nanoparticles used from interfering ions and compounds while maintaining their analytical characteristics.

In this article, we have first proposed a method for increasing the selectivity of metal nanoparticles in the determination of chlorides. The proposed method is based on the simultaneous conversion of chlorine ions to chlorine in situ and its dynamic gas extraction with digital colorimetric detection using reactive indicator papers (RIPs) modified with silver nanoparticles (AgNPs). In contrast to previous studies, the presence of AgNPs provides a sufficiently high sensitivity of the method, and simultaneous dynamic extraction of gases ensures the isolation of chlorine from the effect of the matrix.

## 2. Materials and Methods

### 2.1. Reagents and Solutions

Sodium chloride, sulfuric acid, potassium permanganate, potassium chromate, silver nitrate, fumaric acid, polyvinylpyrrolidone, and sodium hydroxide (chemically pure) were purchased from Sigma-Aldrich (Burlington, MA, USA) and used without further purification. Working solutions of substances were prepared by dissolving their exact weights or aliquots in deionized water. Working chloride standard solutions were prepared by diluting the stock solution immediately prior to use. The sodium chloride solution was standardized by argentometric titration. AgNPs were synthesized according to the procedure described below.

### 2.2. Instrumentation

A CHNOS vario EL cubic analyzer (Elementar Analysensysteme GmbH, Langenselbold, Germany) was used for elemental analysis. An X-Art M energy dispersive X-ray fluorescence spectrometer (Comita, St. Petersburg, Russia) or an MGA-915 atomic absorption spectrometer (Lumex, St. Petersburg, Russia) were used for the determination of silver. A Perkin Elmer Spectrum 100 FTIR spectrometer (Perkin Elmer, Waltham, MA, USA) was used to acquire Fourier Transform IR (FTIR) spectra from KBr pellets using Softspectra data analysis software (Shelton, CT, USA). A STA 409CLuxx synchronous thermal analyzer coupled with a QMS 403CAeolos quadrupole mass spectrometer (NETZSCH, Selb, Germany) and a Perkin-Elmer Diamond TG/DTA derivatograph (Perkin Elmer, Waltham, MA, USA) were used to perform thermal (TA) and differential scanning calorimetric (DSC) analyzes a helium stream (powders, m = 0.3–0.4 g) with the standard α-Al_2_O_3_ at a rate of 2 deg/min in the range of 20–500 °C. Diffractometers DRON-UM-2 (JSC “Burevestnik”, St. Petersburg, Russia), Philips PW 1050 (Philips Analytical X-Ray B.V., Almelo, The Netherlands), and ARL™ X’TRA Powder (Thermo Fisher Scientific, Waltham, MA, USA) with CuKα radiation (λ_Cu_ = 1.54184 Å) were used for X-ray diffraction (XRD) analysis in the range of 2θ = 5–80° with a scanning speed of 5°/min and a temperature of 25 °C. The sizes of crystallites of nanomaterials (D, nm) were determined by the Debye–Scherrer Equation (1):

(1) $$D=\frac{K\lambda }{\beta cos\theta },$$

where *K* is a constant (ca. 0.9); *λ* is the X-ray wavelength used in XRD (1.5418 Å); *θ* is the Bragg angle; and *β* is the pure diffraction broadening of the peak at half-height, that is, broadening due to the crystallite size.

Scanning electron microscopic (SEM) images were taken with a ZEISS Crossbeam 340 device (Carl Zeiss, Jena, Germany) at an accelerating voltage of 3 kV. Secondary electrons were detected with an Everhart-Thornley detector (SE2). X-ray energy dispersive microanalysis (EDX) on an Oxford X-max 80 microanalyzer with an electron probe energy of ≤10 keV was used to determine the distribution of chemical elements on the surface of the samples. A high-resolution transmission microscope Tecnai G2 Spirit BioTWIN FEI (The Netherlands) was used for high-resolution transmission electron microscopy (HRTEM). The deionized water was obtained using the Millipore Simplicity water purification system (Merk Millipore, Burlington, MA, USA). The exact weight of substances was determined on an analytical balance of the 2nd class VLR-20 (Gosmeter, Russia) with an error of ±0.0001 g.

### 2.3. Synthesis of Silver Fumarate

Synthesis was carried out in a room with strongly diffused light or under conditions of illumination with a lamp with a red-light filter. Before use, all chemical vessels were thoroughly washed, rinsed with distilled water, then rinsed three times with small volumes of nitric acid and washed with bidistilled water distilled from a glass distiller. All solutions were prepared on the same bidistillate. Then, 1.16 g of fumaric acid (0.01 mol) was dissolved in 50 mL of a solution containing 0.8 g of NaOH (0.02 mol). In a separate vessel, 3.4 g of AgNO_3_ (0.02 mol) was dissolved in 20 mL of bidistilled water. A solution of sodium fumarate on a magnetic stirrer was added dropwise from a dropping funnel to a solution of silver nitrate at a rate of 20 drops/min. A slight turbidity of the solution and a bluish opalescence were observed at first. As more of the silver nitrate solution was added, the rate of precipitation increased. The resulting suspension was removed from the magnetic stirrer, loosely covered with glass, and left to stand in total darkness for 72 h. After that, with known precautions against exposure to light, it was filtered on a Schott filter (40 pores), washed repeatedly with bidistillate, then dried briefly on the filter and washed with absolute ethanol twice with 30 mL portions. The precipitate was dried in air in the dark for 12 h and dried in a vacuum for 8 h at 80 °C. Dry salt was stored in a brown glass bottle wrapped in black photographic paper; 2.85 g of a finely crystalline salt with a color of white to pale yellow was obtained, which corresponded to a yield of 86.3%. Elemental analysis found %: C—14.62; H—0.683; Ag—64.8; calculated for C_4_H_2_O_4_Ag_2_ %: C—14.54; H—0.6; Ag—64.45.

### 2.4. Preparation of the Nanocomposites

To obtain nanocomposites, the following procedure was used: a sample of silver fumarate (0.8 g) was placed in a quartz tube (height of 10 cm and 2.8 cm in diameter), which in turn was placed in a quartz tube sealed at one end (30 cm in length and 6 cm in diameter). The assembled device was evacuated to a residual pressure of 6 mm Hg and was filled with nitrogen (99.99%) through a hydraulic seal, which used silicone oil as a sealing fluid. The assembled device was then heated to a temperature of 400 °C at a heating rate of 5°/min. Nitrogen and thermolysis products were pumped out, creating a residual vacuum of 4–6 mm Hg, and were kept in a dynamic vacuum at a given temperature for 1 h. The heating was then turned off and they were left to reach room temperature in a dynamic vacuum. The products were removed in the form of a porous column 20–25 mm high and were then crushed. The result was 0.6 g of a black powder. To impregnate the paper, the silver salt thermolysis product was dispersed in water using ultrasound, and the resulting dispersion system was stabilized by adding 0.03% polyvinylpyrrolidone. The resulting dispersion was stable for 1 month.

### 2.5. Technique for Obtaining Papers Modified with AgNPs

For preparation of RIPs, several types of papers with distinctive characteristics were selected, which are shown in Table 1.

AgNPs were deposited on paper using two methods: dropping and immersion.

The dropping (d) of 0.13 mL of a solution of AgNPs with a concentration of 22.3 µg/mL was carried out with a microdosing device on paper samples in a Petri dish. Then, the Petri dish was placed in an oven and dried in air at a temperature of ~80 °C. The operation was performed once or twice.

Impregnation of paper using the immersion (i) method was carried out using the following procedure: 1.5 mL of a solution of AgNPs of various concentrations was applied to a Petri dish and a paper sample 6 cm × 7 cm in size was placed on top of it. This led to the rapid and complete absorption of the AgNPs solution by the paper sample. The samples were placed in an oven and dried in air at a temperature of ~80 °C, horizontally (marked h) or vertically (marked v).

The resulting RIP samples modified with AgNPs are presented in Table 2.

The resulting RIP samples were cut into 0.5 cm × 2.0 cm rectangles and used as test strips. Studies have shown that RIPs are stable for 3 months when stored in a dark bottle with a tightly closed lid.

To study the interaction of chlorine with AgNPs in a paper matrix, diffuse reflection was measured, and the samples were scanned from two sides: from the front side of the application (marking F) and the reverse side (marking R).

### 2.6. Procedure of Analysis of Chloride Ions

**Figure 1:**
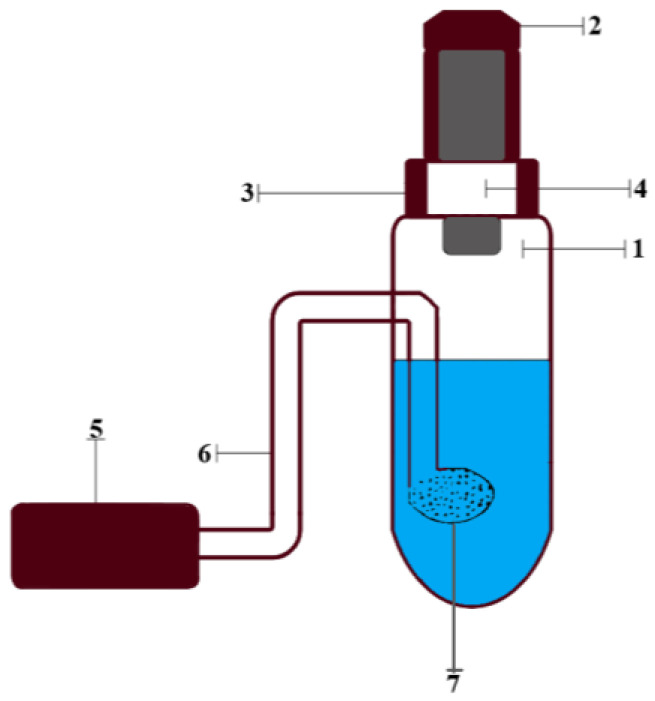
Device for processing dynamic gas extraction: 1—glass container for the analyzed solution; 2—rubber stopper; 3—test strips holder; 4—test strip; 5—air microcompressor, 6—polymer hose; 7—glass bubbler.

Working solutions of the substances were prepared by dissolving their weighed portions and through dilution in deionized water. The stock solution of chlorides (10 mg/L) was prepared by dissolving 0.0082 g of sodium chloride in water in a volumetric flask with a capacity of 500.0 mL. A series of standard chloride solutions were prepared from the stock solution by diluting the appropriate aliquots. The working standard solutions were placed in a setup for dynamic gas extraction, the main components of which are shown in Figure 1: a glass container for the analyzed solution (1) closed with a rubber stopper (2), a test strip holder (3) with a test strip inside (4), an air microcompressor (5) connected by a polymer hose (6) to a glass bubbler (7) inside the vessel. Then, a solution of potassium permanganate and concentrated sulfuric acid were added into a glass container (1). After that, the microcompressor (Hailea Aco-6601) was turned on. After completion of the reaction, the test strips were removed and scanned with a Canon CanoScan LiDE 210 (Canon, Tokyo, Japan) against a white background at 300 ppi. Further, these images were processed in the Adobe Photoshop 7.0 graphics editor in RGB mode by averaging the corresponding color coordinates of individual pixels in a circular reactive zone.

### 2.7. Calibration

To prepare a series of standard solutions, an appropriate portion of the initial stock chloride solution (10 mg/L) was placed in a volumetric flask with a capacity of 100.0 mL and was diluted to the mark with distilled water. In this case, solutions were obtained with the following concentrations of chlorine ions: 0; 0.15; 0.3; 0.6; 1.2, 1.8, and 2.4 mg/L. The standard solutions were sequentially placed into a glass vessel of the dynamic gas extraction unit. Then, concentrated sulfuric acid (2 mL) and 0.2 M potassium permanganate solution (10 mL) were added to that glass vessel. The vessel was tightly closed with a rubber stopper, inside which was the RIPs holder. An air microcompressor was turned on and air was passed through the solution at the optimum volumetric velocity for 20 min. The RIPs were then removed and scanned. At the final stage, the resulting image was processed in RGB (red-green-blue) color coordinates.

### 2.8. Analysis of Samples

Determination of chlorides in each analyzed sample was conducted without any additional preparation, except for dilution (to a concentration of Cl^−^, approximately equal to 0.5 mg/L). Then the diluted solution with a volume of 100.0 mL was placed in a glass vessel (1) (see Figure 1). The analysis was carried out according to the scheme from the previous section.

### 2.9. Analysis of Samples of Natural Water

The analysis of each studied sample was conducted without the stage of sample preparation. The exception was the samples, which had to be diluted to a concentration of Cl^−^ close to 0.5–1.0 mg/L. A diluted 100 mL solution was placed in a glass vessel of a dynamic gas extraction unit and was determined as described in the section above.

## 3. Results and Discussion

### 3.1. Synthesis and Characterization of Silver Fumarate

**Figure 2:**
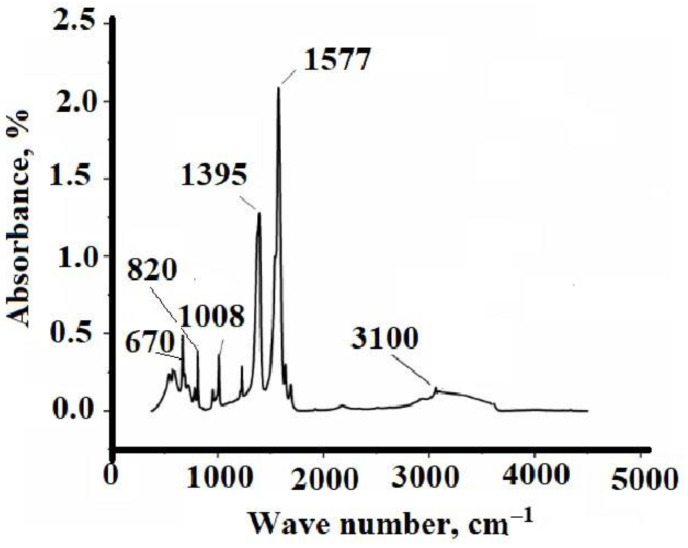
IR spectrum of silver fumarate.

In the present work, silver fumarate was obtained by direct reaction of silver nitrate with fumaric acid in water in an alkaline medium. FT-IR, TGA, and DSC were first employed to confirm the structure and physical phase of silver fumarate. The IR spectrum of silver fumarate (Figure 2) shows several bands of different intensities. In the region of 3100 cm^–1^, an insignificant absorption band is observed, corresponding to the stretching vibrations of the carbon atom, which is in the state of sp^2^ hybridization, connected to the hydrogen atom. A similar band is recorded in the IR spectrum of fumaric acid, but it is slightly shifted to the short wavelength region. Bands at 1395 and 1577 cm^–1^ relate to symmetric and asymmetric vibrations of the carboxyl group. The obtained value Δν is equal to 182 cm^–1^, which may indicate a bidentate mode of coordination of the metal-carboxyl group bond (C_2ν_ symmetry). The band in the region of 1008 cm^–1^ (for fumaric acid it is in the region of 1010 cm^–1^) corresponds to the out-of-plane bending vibrations of the –CH = CH– bonds characteristic of trans isomers. The absorption band in the region of 820 cm^–1^ is similarly characterized, while the band in the region of 670 cm^–1^ is characteristic of the Ag–O bond.

**Figure 3:**
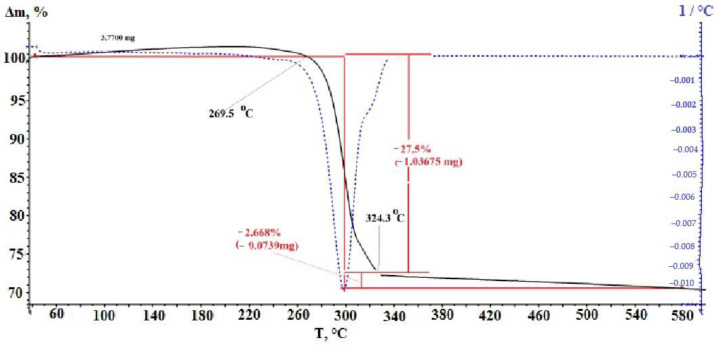
TGA-DTA curve for silver fumarate.

The thermal behavior of silver fumarate was studied (Figure 3). In accordance with the obtained thermogravimetric analysis curve, it can be stated with certainty that the thermal destruction of silver fumarate proceeds in two steps. The first stage begins when the temperature reaches 269.5 °C and ends when 324.3 °C is reached. The TGA curve shows that it is precisely this temperature interval that accounts for the greatest loss of the initial weight of the sample taken for analysis. It can be assumed that decarboxylation occurs at this stage, coupled with the polymerization of the resulting product. The weight loss in this temperature range is 27.5%, which agrees satisfactorily with the theoretical calculation for decarboxylation of the sample (26.6%). At the second stage of thermal transformation, the weight loss is not so pronounced, and the weight gradually decreases in the temperature range from 325 to 580 °C and amounts to about 2.7%. In this case, the intensity of weight loss is insignificant, which is confirmed by the TGA curve.

**Figure 4:**
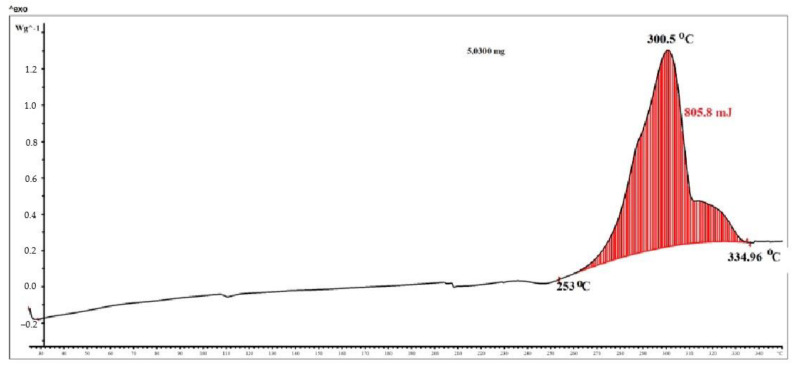
DSC curve of silver fumarate.

The DSC curve (Figure 4) also shows only one exothermic peak, which has a maximum of 300.5 °C, starting at 253 °C and ending at 335 °C. It was in this interval that a significant weight loss occurred. Based on the results of the analysis of DSC, TGA, and DTA, it can be assumed that two processes occur almost simultaneously in the analyzed temperature range: decarboxylation and conjugated polymerization [38]. Decarboxylation is an endothermic process, but the exothermic effect is weakly expressed due to rapid heating (10°/min) followed by polymerization, which has a significant exothermic effect.

The main physicochemical characteristics of the synthesized silver fumarate are consistent with the previously obtained results [39,40].

The mechanism of thermolysis of dicarboxylates is like the mechanism of the thermolysis of salts of unsaturated monocarboxylic acids, which is quite well studied. It can be assumed that, with an increase in the level of thermal vibrations in the monomer lattice, biradicals •OOCCH=CHCOO• are formed, as in this case from fumarate ions. The resulting biradicals react with metal-containing fumarate fragments to form an acid and an H-depleted radical R• of fumarate groups according to the following scheme:

•OCCH=CHCOO• + RH → HOOCCH=CHCOOH + R^I^.

Here, RH = CH_2_CHCOOM(CH_2_=CHCOO)_n−1_, R^I^ = C•HCHCOOM(CH_2_=CHCOO)_n−1_.

**Figure 5:**
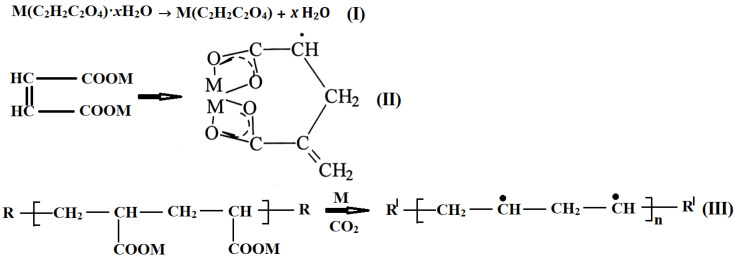
Proposed mechanism of thermolysis of metal fumarates.

The resulting R^I^ can open the double bond intramolecularly (**II**, Figure 5). This is followed by the process of decarboxylation and polymerization, which can be expressed by the scheme (**III**), shown in Figure 5.

### 3.2. Thermolysis of Silver Fumarate

**Figure 6:**
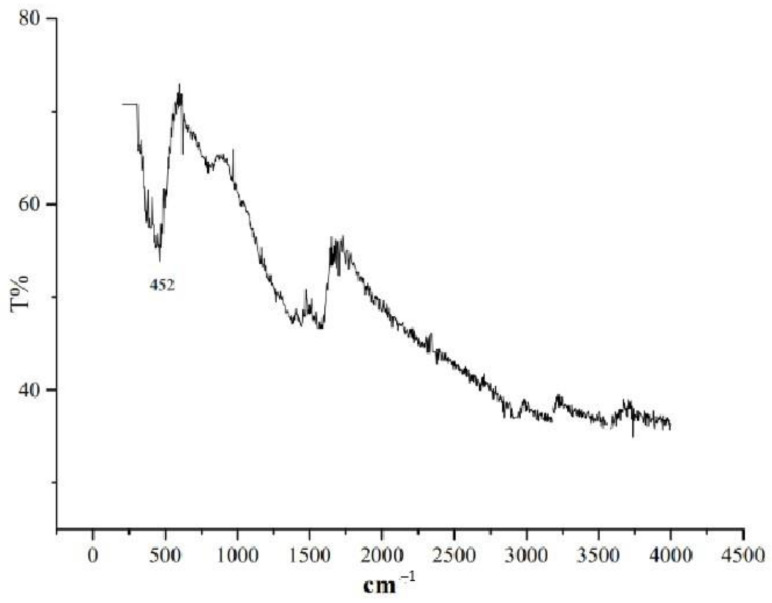
IR spectrum of the thermolysis product of silver fumarate.

As a result of thermolysis of silver fumarate, a black-brown powder was obtained. The color is easily explained by the presence of amorphous carbon [41]. In the IR spectrum of the synthesized substances (Figure 6), no characteristic bands are traced except for the band at 452 cm^–1^, which is characteristic of vibrations of C–H bonds.

**Figure 7:**
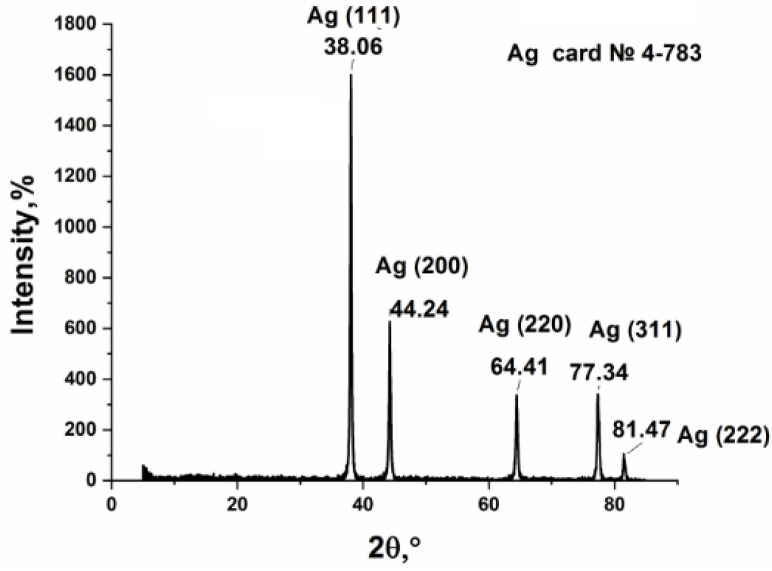
XRD profile of the silver fumarate thermolysis product.

The phase composition of the thermolysis product (Figure 7), which is nanosized silver particles, was studied (JCPDS file 4-783). The average crystallite size calculated by the Debye–Scherrer formula is estimated to be 20.54 nm.

**Figure 8:**
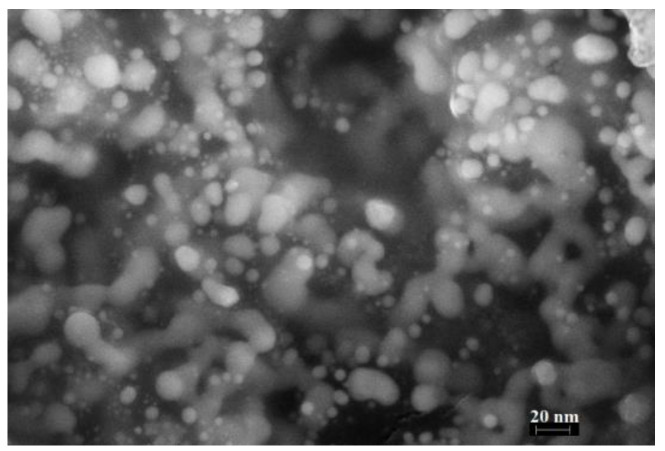
SEM image of the silver fumarate thermolysis product.

The SEM image (Figure 8) visualizes spherical silver particles with sizes from 4 to 22.6 nm against the background of a small amount of carbon-containing material. In addition, aggregated silver particles are detected.

**Figure 9:**
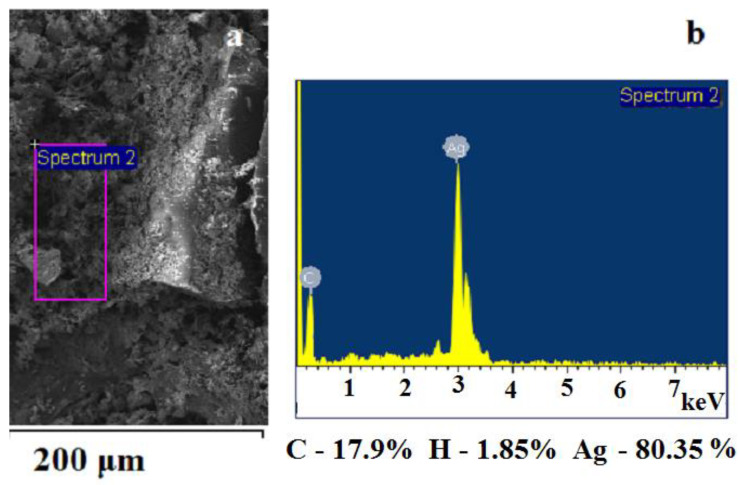
(**a**,**b**) Elemental composition of the silver fumarate thermolysis product according to EDX data.

The elemental composition of the thermolysis products was determined from the data of the energy dispersive (Figure 9) and chemical analyses. The convergence of the results is satisfactory (Table 3).

**Figure 10:**
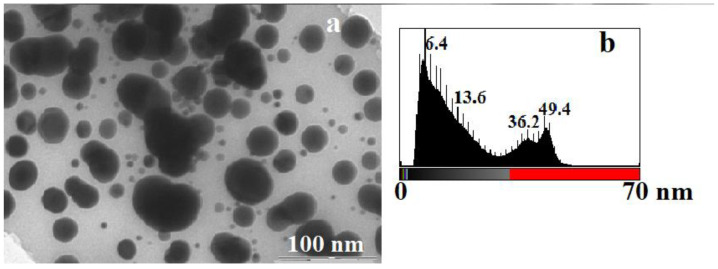
TEM image (**a**) of the silver fumarate thermolysis product and (**b**) histogram illustrating the particle size distribution in the silver fumarate thermolysis product.

Separate, mostly spherical silver particles with sizes from 4.8 to 51.3 nm are visualized in the TEM image (Figure 10a) of the thermolysis product of silver fumarate. The most common particles (Figure 10b) are 6.4 nm in size. The TEM image also shows that the largest particles are either aggregates of several particles or are visualized as an overlay of several particles (areas with high optical density).

**Figure 11:**
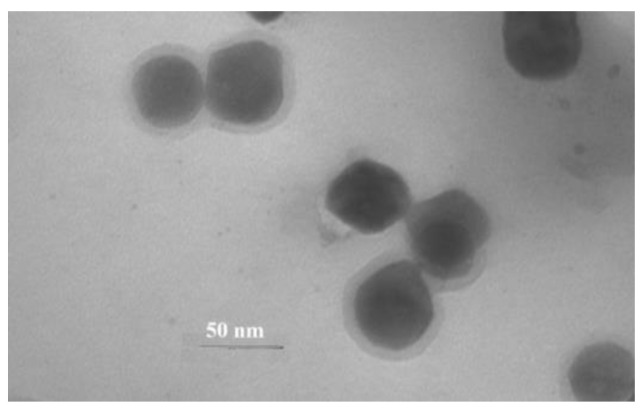
TEM image of the product of thermolysis of silver fumarate by ultrasound in an aqueous medium after 7 min of the start of treatment.

To obtain a dispersion of AgNPs and subsequent impregnation on test strips, the product of thermolysis of silver fumarate in an aqueous medium was treated with ultrasound with a power of 800 W at a frequency of 22 kHz for 20 min at a temperature of 60–70 °C. After 7 min of the treatment of the thermolysis product, swelling of the carbon shell surrounding AgNPs is noted, which is visualized in the TEM image (Figure 11).

**Figure 12:**
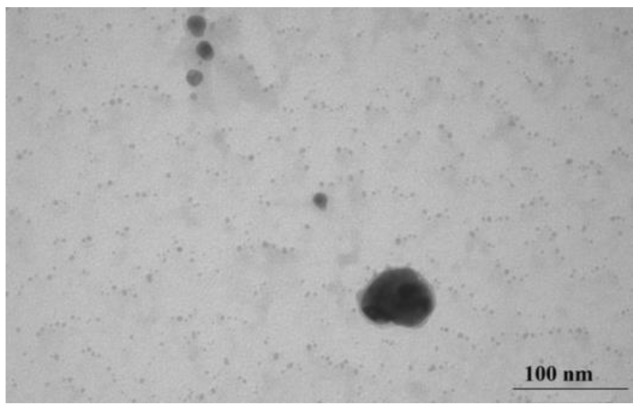
TEM image of the product of thermolysis of silver fumarate in an aqueous medium by ultrasound after 20 min of treatment.

Twenty minutes after the start of processing, only single large particles remain in the field-of-view of the TEM image (Figure 12), while the bulk of the particles are 3.2–3.8 nm in size.

Finally, polyvinylpyrrolidone was introduced into the resulting colloidal solution in an amount of 0.03 wt.% to stabilize the AgNPs.

### 3.3. Preparation of RIPs and Their Characterization

In this work, to obtain RIPs, it is proposed to use the impregnation method. This method is characterized by simplicity and speed, while allowing a large amount of reagent to be deposited on the matrix, which favorably distinguishes it from other methods, such as, for example, sorption modification [42].

**Figure 13:**
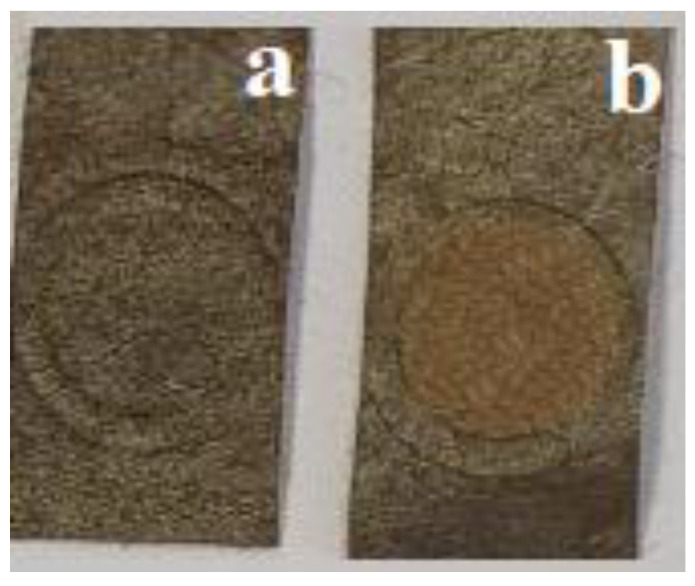
Photos of RIPs before and after interaction with chlorine. (**a**) original sample, (**b**) after treatment with chlorine.

The obtained RIP samples have a gray color with a silver tint, which turns beige when treated with chlorine gas (Figure 13).

To optimize the RIP manufacturing method, the sensitivity to chlorine and the reproducibility (or repeatability) of the characteristics of the modified papers within the same sample and for different samples within the same batch were considered as the main criteria. Sensitivity was evaluated by the diffuse reflectance spectra and the difference in color coordinates (Δy) of the reaction zone of the test strips before (y_0_) and after interaction with chlorine y(Cl^−^) obtained in the reaction system from a chloride solution with a concentration of 0.6 mg/L: Δy = y(Cl^−^) − y_0_.

During an experimental study, the influence of the type of paper, the method of impregnation, and the drying conditions of modified papers, as well as the number of AgNPs deposited on the sample, was studied.

**Figure 14:**
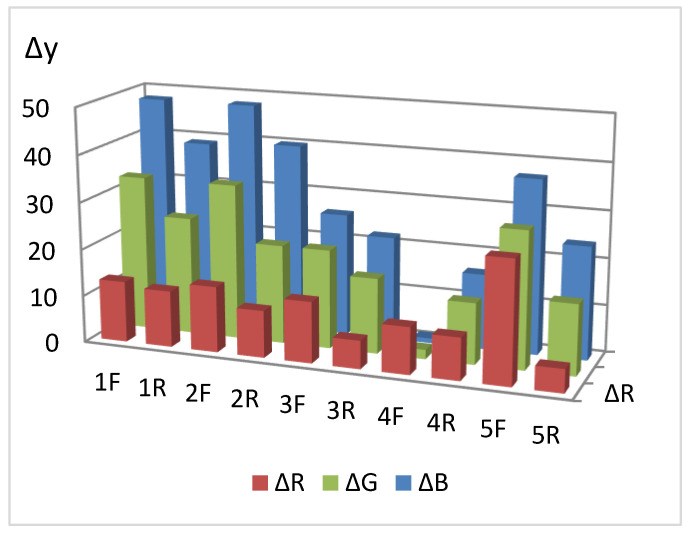
Comparison of types of paper to produce RIPs by single dropping.

Figure 14 shows a diagram for various RIP samples obtained by single dropping (samples **1**–**5**), from which it follows that the maximum values of Δy are observed for type **A** and type **B** papers.

**Figure 15:**
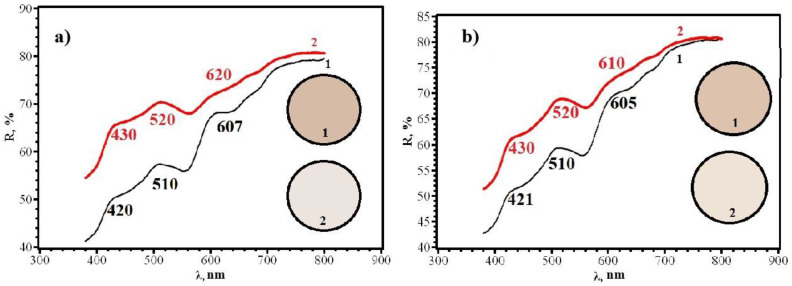
Diffusion reflectance spectra of samples **2F** (**a**) and **2R** (**b**) before (1) and after (2) contact with chlorine.

Comparison of Δy for different sides of RIPs shows that, for all types of papers modified by single dropping, the front side (application side) is more sensitive. This conclusion is confirmed by the diffuse reflectance spectra on the example of paper **B** (Figure 15), which differ significantly for the front and back sides. This circumstance may be due to the limited penetration of AgNPs through paper and their faster adsorption on the side from which the solution is applied and should be considered when working with paper as a test agent.

**Figure 16:**
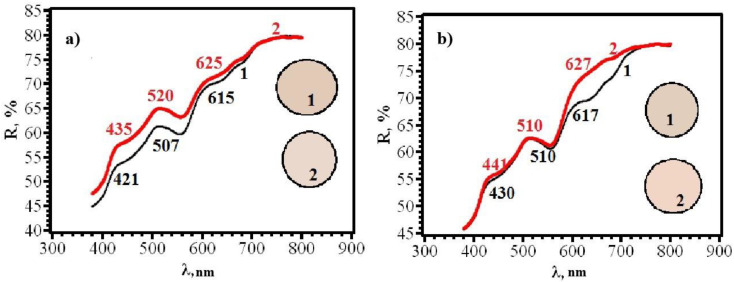
Diffusion reflectance spectra of samples **8F** (**a**) and **8R** (**b**) before (1) and after (2) contact with chlorine.

**Figure 17:**
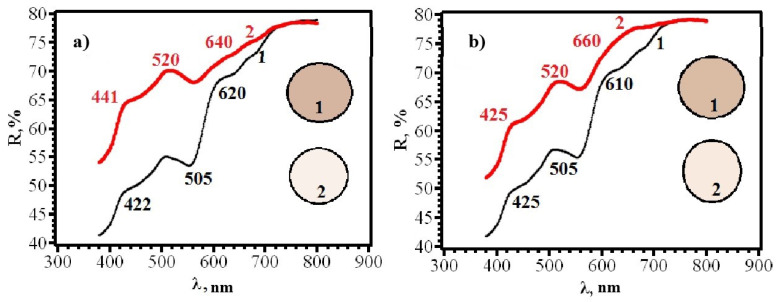
Diffusion reflectance spectra of samples **9F** (**a**) and **9R** (**b**) before (1) and after (2) contact with chlorine.

**Figure 18:**
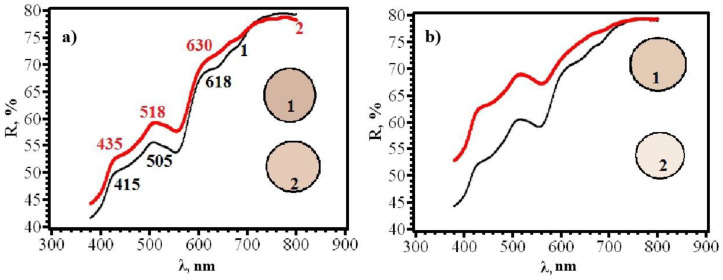
Diffusion reflectance spectra of samples **10F** (**a**) and **10R** (**b**) before (1) and after (2) contact with chlorine.

**Figure 19:**
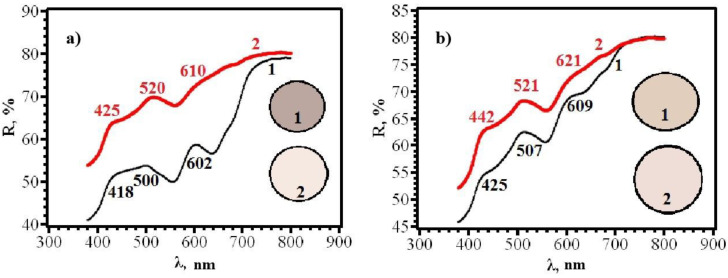
Diffusion reflectance spectra of samples **11F** (**a**) and **11R** (**b**) before (1) and after (2) contact with chlorine.

Similar results were obtained for papers of types **A** and **B**, from which RIPs were made using immersion (Figure 16, Figure 17, Figure 18 and Figure 19).

**Figure 20:**
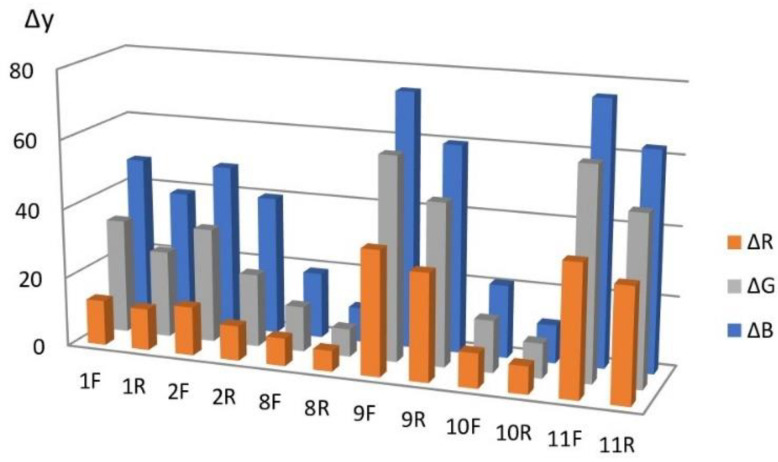
Comparison of RIPs obtained by impregnation and drying methods.

For samples **8**–**11**, the content of AgNPs deposited on paper in this way was 0.33 mg/g, which is comparable to the content of particles on RIP obtained by a single drop. This made it possible to compare different impregnation methods (Figure 20). In addition to the impregnation method, drying methods were compared in the preparation of samples. Samples **8** and **10** were fixed in clamps and dried vertically and samples **9** and **11** were dried horizontally on a Petri dish in which impregnation was carried out. It should be noted that, previously [43], samples were dried in air at room temperature. It required a lot of time. In this paper, it is proposed to dry RIP samples at a temperature of ~80 °C. This method allows you to quickly obtain many samples of modified papers in a relatively short time. In addition, the data obtained indicate the thermal stability of AgNPs under these conditions.

The data presented in the comparative diagram allow us to conclude that the maximum sensitivity is observed:

- When using papers of **A** and **B** types, which makes it possible to recommend them for use as AgNP cellulose carriers for the manufacture of chlorine-sensitive RIPs;
- In the manufacture of RIPs by immersion followed by horizontal drying. Visual control of the surface of the samples during the study showed that it is in this case that the most uniform modification of the surface of large samples is achieved. However, the dropping method is a simpler procedure and can be quite effective in the case of small samples;
- When scanning test strips from the front.

In addition, based on the diagram, it can be assumed that, for these RIP samples, the blue coordinate of the RGB system will be the most sensitive.

As is known, the spectral characteristics of RIP samples and the intensity of their coloration are affected by the content of nanoparticles [22,24,42,44,45,46,47,48,49]. This point is important from the point of view of chemical analysis, since it is well known that the color intensity of any test strip, as well as the content of the reagent in it, has a strong influence on the metrological characteristics of the determination, such as the limit of detection (LOD) and the limits of quantification (LOQs). Therefore, the next stage of our study was to investigate the effect of the content of AgNPs on the sensitivity of RIPs to chlorine.

Using the selected types of papers (**A** and **B**), RIPs were made by double dropping (samples **6** and **7**). In the manufacture of RIPs by double immersion, samples were obtained with an uneven distribution of AgNPs over the surface. Therefore, in this case, we had to conduct a single immersion, increasing the concentration of nanoparticles in the solution for impregnation (samples **12**–**17**).

**Figure 21:**
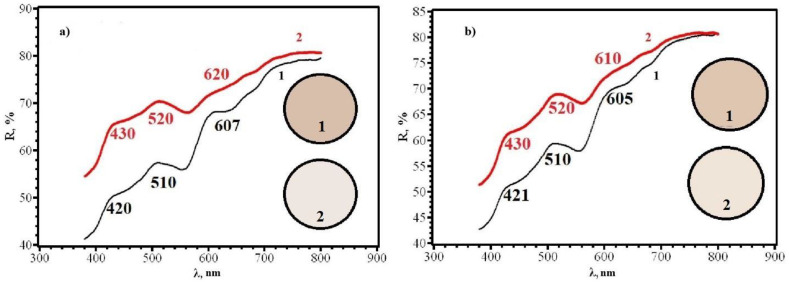
Diffusion reflectance spectra of (**a**) **6F** and (**b**) **6R** papers before (1) and after (2) contact with chlorine.

**Figure 22:**
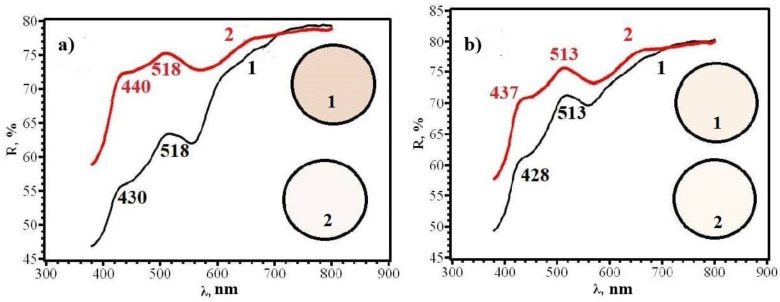
Diffusion reflectance spectra of samples **7F** (**a**) and **7R** (**b**) before (1) and after (2) contact with chlorine.

**Figure 23:**
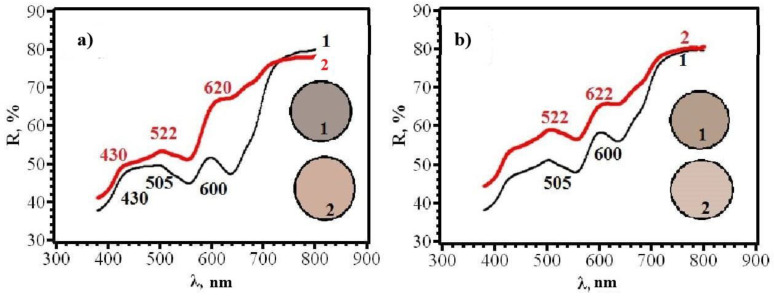
Diffusion reflectance spectra of samples **12F** (**a**) and **12R** (**b**) before (1) and after (2) contact with chlorine.

**Figure 24:**
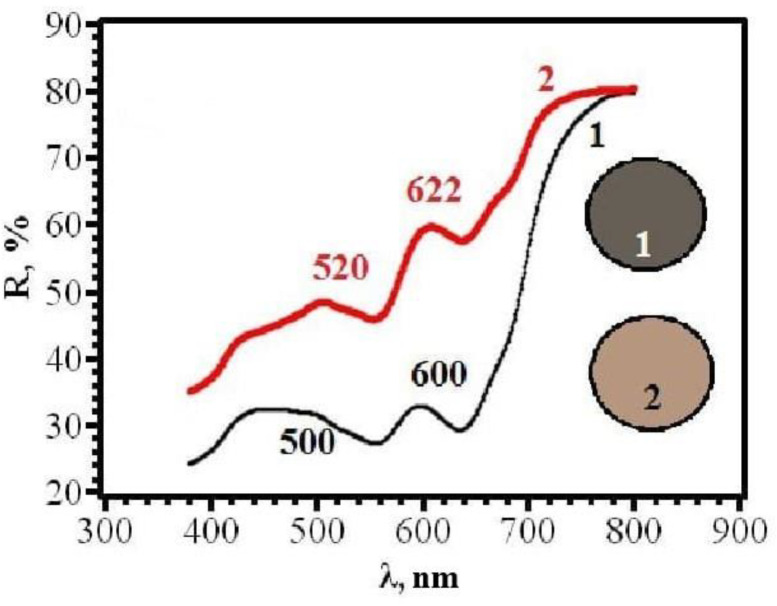
Diffusion reflectance spectra of samples **14F** before (1) and after (2) contact with chlorine.

**Figure 25:**
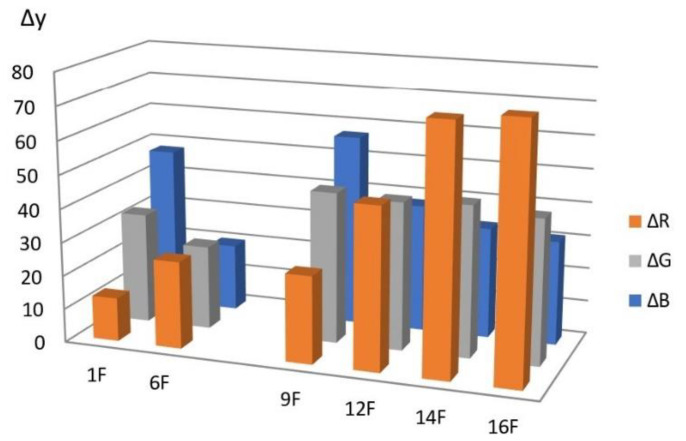
Influence of the content of AgNPs on the sensitivity of RIPs to chlorine.

With an increase in the content of AgNPs, an increase in the intensity of the gray color of RIPs is observed. The transition to beige after contact with chlorine becomes more contrasting. At the same time, it was found that the front side of RIP is still more sensitive, both for samples obtained using dropping (Figure 21 and Figure 22) and for samples obtained using immersion (Figure 23 and Figure 24). Therefore, in the further study, only the reagent application side (**F**) was considered (Figure 25). The change in the content of AgNPs did not affect the choice of the impregnation method. RIPs obtained using immersion are still the most sensitive to chlorine.

**Figure 26:**
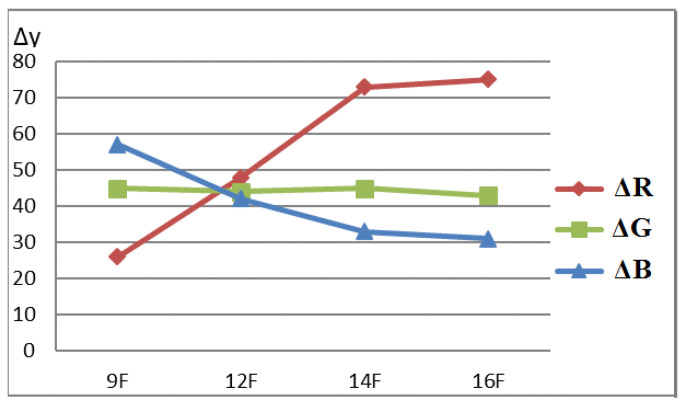
Dependence of the sensitivity of color coordinates on the AgNP content on the RIP surface.

It can be seen from the comparative diagram that, with an increase in the content of nanoparticles, a gradual change in the sensitivity of color coordinates to chlorine occurs. If, for samples with a low AgNPs content, the maximum value of Δy is observed for the blue coordinate, then with an increase in AgNPs content, the red coordinate becomes the most sensitive. Most clearly, the trend of change can be demonstrated in the form of graphs in Figure 26. Obviously, to increase the sensitivity of RIPs to chlorine, the content of AgNPs should be increased to 0.62 mg/g. A further increase does not lead to significant changes and may be due to the aggregation of nanoparticles in a high concentration solution. The dependence of the sensitivity of the R-coordinate on the content of AgNPs in the range from 0.24 mg/g to 1.85 mg/g is almost linear, which allows us to recommend the proposed methodological approach to obtaining the most effective test tools with specified analytical characteristics.

Thus, because of the conducted studies, the following parameters were selected for the manufacture of chlorine-sensitive RIPs:

- Paper type (**A** or **B**);
- Method of impregnation (immersion);
- Content of AgNPs 0.62 mg/g;
- Scanning from the side of application of AgNPs.

In addition, it was assumed that, when using such RIP samples for the determination of chloride ions, the R-coordinate of the RGB system will be the most effective analytical signal.

For samples **14** and **15**, obtained using the optimized method, a quantitative assessment was made of the uniformity of the distribution of AgNPs over the paper surface and the reproducibility of applying the reagent. To do this, the values of the relative standard deviation (RSD) of the measurement of diffuse reflectance at 610 nm, R_610_, were calculated for different surface areas within the same sample and for different samples within the same batch. It was found that the RSD of R_610_ is 0.024–0.044 for sample **14** and 0.031–0.041 for sample **15**. Satisfactory values of s_r_ indicate the possibility of using the obtained RIPs for the manufacture of test strips. Further studies were conducted with sample **14**.

### 3.4. Determination of Chlorides

The technique includes the following steps: oxidation of chloride ions in solution (2Cl^−^ + Ox = Cl_2_ + Red), gas extraction of chlorine, its interaction with AgNPs fixed on the RIP surface (Cl_2_ + 2Ag^0^ = 2AgCl), and determination of the color intensity of the reaction zone test strip, which changes depending on the concentration of chlorides in the solution.

**Figure 27:**
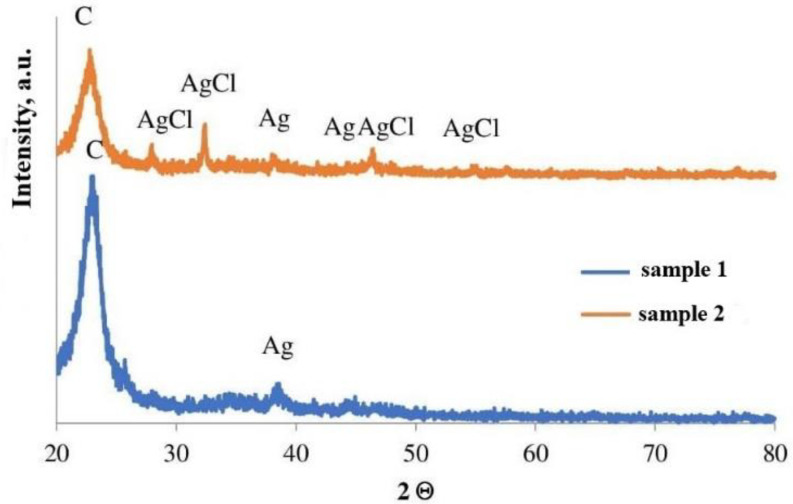
XRD patterns of the reaction zone of RIPs before (sample 1) and after (sample 2) interaction with chlorine.

The formation of silver chloride was confirmed using XRD (Figure 27).

An important feature of gas extraction is the spatial separation of AgNPs from interfering ions and other nonvolatile compounds by an air barrier. Conducting a color reaction outside the sample ensures the selectivity of the analysis, and the dynamic method of extraction significantly increases the sensitivity of the determination.

### 3.5. Optimization of Determination Conditions

The determination conditions (acidity, gas extraction time, air supply rate, etc.) are optimized. It has been experimentally established that the optimal air flow rate is 2.8–3 L/min. An increase in rate leads to a violation of the clear boundary of the reaction zone, and a decrease leads to an increase in the analysis time. When choosing the acidity and extraction time, the degree of extraction (DE) was determined by the formula: DE = (C_0_ − C)100/C_0_, %, where C_0_ is the initial concentration of chlorides, C is the concentration of chlorides obtained after extraction. In practice, it has been confirmed that to suppress the hydrolysis of chlorine, the most acceptable option is to acidify the sample to pH 0–1 by adding concentrated sulfuric acid (2 mL) to 100 mL of the solution (Figure 19a). The analysis of the dependence of DE on the time of gas extraction presented in Figure 19b) indicates almost complete recovery of chlorides after 20 min from the start of the experiment. The optimal sample volume is 100 mL for most analyzed objects. Provided that the chloride content in the sample exceeds the upper limit of the range, a smaller volume should be taken for analysis, diluting it to 100 mL with distilled water.

To completely oxidize chloride ions, any non-volatile oxidizing agent whose potential is greater than 1.36 V can be used, for example, potassium permanganate (1.51 V). However, it should be considered that, when using it, the determination of chlorides will be interfered with by bromides and iodides in the ratio of 1:1 [49,50], which leads to significant limitations in the use of the technique. Namely, it cannot be used in the analysis of mineral, marine, and associated formation waters without additional sample preparation. In addition, molecular chlorine, bromine, and iodine can cause the discoloration of test strips. Accordingly, if their presence is established, the sample must first be degassed. It should be noted that non-volatile compounds that do not form gaseous substances in an acidic environment do not affect the color of the test strips. This circumstance indicates the possibility of determining chlorides in various complex multicomponent objects.

LOQs were established experimentally using standard model solutions of chlorides. The difference in the color of the reaction zone of the test strips during the analysis under the selected conditions is observed in the range from 0.15 to 2.4 mg/L (Table 4). A simple and accessible method of colorimetry was used to describe the coloration. Using scanner technologies and image processing software, color coordinates were obtained in the RGB system. Table 4 presents the average coordinates of five parallel measurements.

The process of color change in the reaction zone of the test strip leads to an increase in all three-color components. To select the most sensitive and promising analytical signal, the dependences of R-, G-, B-coordinates on the concentration of chlorides are described using exponential equations of the form y = y_0_ + A(1 − exp(−c(X)/t)), the regression parameters of which y_0_, A and t are presented in Table 5.

**Figure 28:**
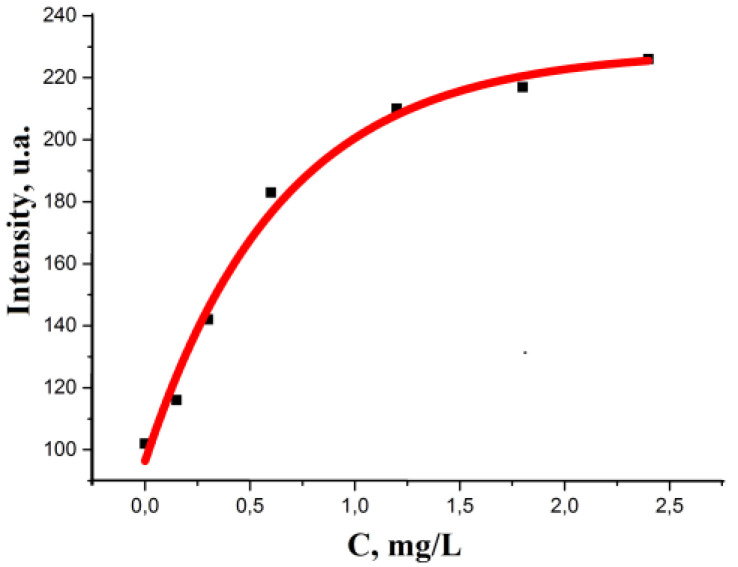
Changes in R-color coordinate of Ag NPs-modified paper on the concentration of chloride ions in the analyzed solution.

Considering that the A/t value is a criterion for choosing the optimal color coordinate since the larger its value, the lower the LOD; then, for the colorimetric determination of chlorides according to the proposed method, the red coordinate R should be selected as an analytical signal. A view of the calibration curve on the example of chlorides is presented in Figure 28. For convenience, the exponential dependence can be translated into a linear form: −ln((y_0_ − y)/A + 1) = c(Cl^−^)/t and is described by the equation Y = 0.9X (R^2^ = 0.9925), where Y = −ln((y_0_ − y)/A + 1), X = c(Cl^−^)/t.

As a result of comparing the A/t values for these methods, it can be concluded that the developed method is not inferior in sensitivity to those described earlier. This conclusion is in good agreement with the LOD calculated by the 3 s criterion, which is 0.036 mg/L.

### 3.6. Interference Study

According to the proposed approach, non-volatile compounds, which do not produce gaseous substances in an acidic medium, should not affect the AgNPs on the paper. This was confirmed by determination of a known amount of chloride (0.6 mg/L) in the presence of 1 M solutions of salts of some common inorganic ions. The results are represented in Table 6. They indicate that relative errors lower than 7% are produced.

However, volatile compounds in acidic environments (chlorine, bromine, iodine, hydrogen sulfide, and acetic acid), or substances that form volatile oxidizing agents when KMnO_4_ is added (bromides, iodides), can have a noticeable effect on AgNPs in a 1:1 ratio. Therefore, the technique cannot be applied to the analysis of objects containing these substances without an additional stage. In this case, potassium dichromate should be added to the solution in the bubbler and gas extraction should be carried out, during which acetic acid, dissolved gases, and gases formed from salts will be removed from the solution. Only after this procedure should potassium permanganate be added to the solution and then chlorides can be determined.

The color of the analyzed objects does not interfere with the determination, since the reaction mixture and the test strip, on which the color reaction of AgNPs with chlorine proceeds, are separated by air space. This significantly increases the selectivity of the determination and expands the scope of the technique. Determination of chlorides can be carried out in colored natural waters, pharmaceutical preparations with the addition of dyes and herbal extracts, food products containing tomato, and colored food additives.

### 3.7. Determination of Chlorides in Natural Waters

For the purposes of hydrochemical analysis, testing was conducted on water samples from the Don River (Rostov-on-Don, Russia), in which, according to the literature data [51], the chloride concentration is 120–140 mg/L and the bromide content is less than 0.001 mg/L. To check the reliability of the results of the analysis, argentometric titration according to the Mohr method was used as a control method. Based on the average concentration of chlorides in the analyzed object and considering the working range of the developed method, the volume of the aliquot was 0.5–1.5 mL, i.e., 100 times less than for titrimetric determination.

The results obtained are presented in Table 7. Comparison by Fisher’s test indicates that there is good convergence between the results obtained by the two methods. Precision is shown as the RSD of the results of a single analysis, obtained using the method under repeatability conditions. The correctness of the developed methodology was confirmed using the “introduced-found” method. The addition of an analytical standard of a pure substance with a known value (“introduced”) to the test sample showed that the increase in the analytical signal corresponds to the introduced additive (“found”).

### 3.8. Determination of Chlorides in Pharmaceutical Preparations

The developed method is a suitable alternative to quality control methods for pharmaceutical preparations containing ionically bound chloride ions. The results presented in Table 8 demonstrate the capabilities of the method and show the convergence with the control method and the absence of interfering influence of concomitant substances.

In the analysis of preparations, the developed method is more economical due to the reduction in the cost of silver nitrate, but its main advantage is its high sensitivity (4 × 10^–6^ mol/L) and selectivity in comparison with titrimetry. This opens wide opportunities for using the technique for the purposes of toxicological chemistry, pharmacokinetics, and the diagnosis of diseases such as cystic fibrosis.

### 3.9. Determination of Chlorides in Foods

Salt content is one of the mandatory standardized indicators for food products, including canned fish. Control is conducted both in technological processes and in finished products in accordance with Russian GOST 27207-87 “Canned food and preserves from fish and seafood”. The method currently used for the determination of the salt involves the use of argentometric titration according to the Mohr method. Despite the fact that titration itself is a fairly fast analysis, sample preparation takes a long time and consists of grinding the product, preparing an aqueous extract, filtering, and neutralizing it and, in the case of analyzing canned fish in tomato sauce, salt determination is carried out with preliminary sample ashing. As a result, the time taken for a single determination reaches from two to several hours.

When using the developed extraction-colorimetric technique, it is required to grind the sample and, in the case of analyzing canned food in marinade, eliminate the interfering effect of acetic acid by preliminary gas extraction without adding potassium permanganate. As a result, the analysis time is reduced by five to six times due to the minimization of the sample preparation stage. In addition, reagent costs are reduced by eliminating costly silver nitrate.

The results obtained using the developed and control (Russian GOST 27207-87) methods are presented in Table 9. Comparison by the Fisher criterion indicates that there is good convergence between the results obtained by the two methods. Precision is shown as RSD of the results of a single analysis, obtained using the method under repeatability conditions.

## 4. Conclusions

Analysis of the data presented shows that thermolysis of silver fumarate leads to the formation of nanocomposites containing metal nanoparticles uniformly distributed in a stabilizing carbon matrix. The nanomaterials obtained are characterized by stability over time; that is, when they are stored for a long time, there are no changes in the chemical composition, size, and shape of the nanoparticles. The main advantages of the proposed method for obtaining nanocomposites are simplicity, softness, and low cost, which allow it to be used for large-scale production. It is established that the size of silver crystallites varies from 2.1 to 32.4 nm. The optical properties of the obtained nanocomposites make it possible to use them to create new reactive indicator papers that change their color depending on the concentration of chlorides in the solution. The method for determining chlorides, based on the oxidation of chlorides and the extraction of the resulting chlorine with an air stream, is completed using digital colorimetric detection and is the basis for obtaining the dependences of the color of test strips on the concentration of chlorides. The use of gas extraction in the developed method makes it possible to divide the space of two-color reactions and increase the selectivity of the determination. Simultaneously carrying out of the processes of chloride oxidation, gas extraction of chlorine, and the reaction of chlorine with the nanocomposites ensures rapid determination, which is another advantage of the method.

## Figures and Tables

**Table 1:** Type of papers for preparing RIPs.

Symbol	Paper Grade	Manufacturer	Paper Specifications
**A**	595	Whatman (Cytiva, Little Chalfont, Buckinghamshire, UK)	Density 62.5 g/m^2^, thickness 0.17 mm, pore diameter 7–11 μm
**B**	597		Density 85 g/m^2^, thickness 0.18 mm, pore diameter 4–7 μm
**C**	598-A		Density 75 g/m^2^, thickness 0.19 mm, pore diameter 5–7 μm
**D**	B1	JSC Goznak (St. Petersburg, Russia)	Density 200 g/m^2^, thickness 0.3 mm, pore diameter 7–11 μm
**E**	FN 18	Filtrak (Filtra acido hydrochlorico extracta) (Spezialpapierfabrik Niederschlag, Germany)	Density 280 g/m^2^, thickness 0.4 mm, pore diameter 7–11 μm

**Table 2:** RIP samples.

Sample	Paper Type	Dropping Method	Immersion Method	Multiplicity of Processing
**1**	**A**	d	h	1
**2**	**B**	d	h	1
**3**	**C**	d	h	1
**4**	**D**	d	h	1
**5**	**E**	d	h	1
**6**	**A**	d	h	2
**7**	**B**	d	h	2
**8**	**A**	i	v	1
**9**	**A**	i	h	1
**10**	**B**	i	v	1
**11**	**B**	i	h	1
**12**	**A**	i	h	2
**13**	**B**	i	h	2
**14**	**A**	i	h	3
**15**	**B**	i	h	3
**16**	**A**	i	h	4

**Table 3:** Elemental composition of the thermolysis product of silver fumarate according to chemical analysis data.

Element	Content, %
C	18.2 ± 0.1
H	1.76 ± 0.23
Ag	80.24 ± 0.37

**Table 4:** Average coordinates of 5 parallel measurements in the RGB system.

mg/L	0.0	0.15	0.3	0.6	1.2	1.8	2.4
	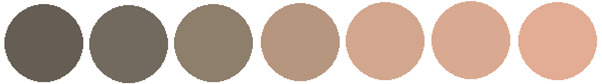						
**R**	102	116	142	183	210	217	226
**G**	94	105	126	150	167	170	173
**B**	85	95	108	128	141	145	148

**Table 5:** Coefficients of exponential equations of color coordinates dependence on chloride concentration.

Color Coordinate	Regression Parameters				
	y_0_	A	t	A/t	R
R	96 ± 6	132 ± 8	0.65 ± 0.12	203	0.9893
G	91 ± 4	84 ± 5	0.54 ± 0.09	156	0.9899
B	83 ± 2	66 ± 3	0.59 ± 0.08	111	0.9939

**Table 6:** Determination of chloride ions in the presence of some common inorganic ions.

Added Cl^−^, mg L^−1^	Ion	Found Cl^−^, mg L^−1^	Relative Error, %	Lower Limit of Tolerance (mol/mol)
0	Each ion	<LOD	-	-
0.6	Na^+^	0.610	+2	1.4 × 10^5^
	K^+^	0.604	+1	7.1 × 10^4^
	Mg^2+^	0.587	−2	7.1 × 10^4^
	Ca^2+^	0.558	−7	7.1 × 10^4^
	Sr^2+^	0.617	+3	7.1 × 10^4^
	Ba^2+^	0.613	+2	7.1 × 10^4^
	Mn^2+^	0.582	−3	7.1 × 10^4^
	Zn^2+^	0.586	−2	7.1 × 10^4^
	NH_4_^+^	0.606	+1	7.1 × 10^4^
	Fe^3+^	0.611	+2	7.1 × 10^4^
	NO_3_^−^	0.565	−6	1.4 × 10^5^
	HPO_4_^2−^	0.605	+1	7.1 × 10^4^
	H_2_PO_4_^−^	0.606	+1	7.1 × 10^4^
	SO_4_^2−^	0.588	−2	7.1 × 10^4^
	C_2_O_4_^2−^	0.584	−3	7.1 × 10^4^
	Cr_2_O_7_^2−^	0.612	+2	7.1 × 10^4^

**Table 7:** The results of chlorides determination using colorimetric and titrimetric methods in water samples of the river Don (Rostov-on-Don) (*n* = 4; *p* = 0.95; *F_theor_* = 9.28; *t* = 3.18).

Sample	Added Cl^−^, mg L^−1^	Found (mg L^−1^) by				*F_theor_*	Recovery (%)
		Proposed Method		Control Method *			
		X_av_ ± δ	RSD (%)	X_av_ ± δ	RSD (%)		
1	-	129 ± 3	1.4	135 ± 2	1.0	1.19	95.6
	5.0	133 ± 4	1.9	140 ± 3	1.3	1.76	95.0
2	-	131 ± 2	1.0	128 ± 2	1.0	1.17	102.3
	10.0	140 ± 7	3.2	136 ± 4	1.9	0.33	102.9
3	-	133 ± 4	1.9	127 ± 3	1.5	1.57	104.7
	30.0	159 ± 5	2.0	161 ± 6	2.3	0.69	98.8

* Control method is Mohr’s argentometric method $\overset{-}{X}$.

**Table 8:** Results of pharmaceuticals analysis (*n* = 5; *p* = 0.95; F*_theor_* = 6.59; t = 2.78).

A Drug/ Substance/ Manufacturer	Concomitant Substances	Found (mg) by				*F_theor_*	Recovery (%)
		Proposed Method		Control Method *			
		X_av_ ± δ	RSD (%)	X_av_ ± δ	RSD (%)		
“Magnesium B6 effervescent tablets”/ pyridoxine hydrochloride (vitamin B6)—1.4 мг C_8_H_11_HO_3_•HCl/ Pez Production Europe Kft, Hungary	citric acid, sodium bicarbonate, sugar, magnesium bicarbonate flavoring, aspartame, yellow dye: sodium salt of riboflavin 5-phosphate	1.45 ± 0.11	6.1	1.39 ± 0.12	6.9	1.21	104.3
«Anaprilin Reneval»/Propranolol hydrochloride—10 mg C_16_H_21_NO_2_•HCl/ Renewal of PFK JSC, Russia	Sucrose, potato starch, calcium stearate, talc	9.4 ± 0.8	6.8	10.2 ± 1.1	8.7	2.27	92.2
“Formetin”/Metformin hydrochloride—500 mg C_4_H_11_N_5_•HCl/Pharmstandard-Leksredstva, Russia	Povidone, croscarmellose sodium (primellose), magnesium stearate	509 ± 21	3.3	491 ± 18	2.9	1.36	103.6

* Control method is acid-base potentiometric titration.

**Table 9:** The results of the determination of salt in canned fish (*n* = 3; *p* = 0.95; *F_theor_* = 19.2; *t =* 4.3).

Canned Fish in Tomato Sauce (Russian GOST 16978-99)	Found ω (%) by						*F_exp_*	Recovery (%)
	Proposed Method (25 min) with Elimination of Acetic Acid (45 min)			Control Method * with Ashing (4.5 h)				
	X_av_ ± δ	Δ, %	RSD (%)	X_av_ ± δ	Δ, %	RSD (%)		
Pink salmon	1.60 ± 0.11	6.9	2.8	1.51 ± 0.12	8.0	3.2	1.31	105.9
Smelt	1.19 ± 0.08	6.7	2.7	1.32 ± 0.12	9.1	3.6	1.78	90.2
Sprat	1.90 ± 0.07	2.6	1.5	2.1 ± 0.2	9.5	3.8	6.42	90.5
Gobies	1.40 ± 0.05	3.5	1.4	1.29 ± 0.11	8.5	3.4	5.90	108.5

* Control method is Mohr’s argentometric method with preliminary ashing of the sample.

## Data Availability

The data presented in this study are available on request from the corresponding author.

## References

[1] Teixeira dos Santos C.A., Pascoa R.N.M.J., Porto P.A.L.S., Cerdeira Lopes J.A. (2016). Application of Fourier-transform infrared spectroscopy for the determination of chloride and sulfate in wines. LWT Food Sci. Technol..

[2] De Oliveira Souza M., Ribeiro M.A., Carneiro M.T.W.D., Athayde G.P.B., de Castro E.V.R., da Silva F.L.F., Matos W.O., de Queiroz Ferreira R. (2015). Evaluation and determination of chloride in crude oil based on the counterions Na, Ca, Mg, Sr and Fe, quantified via ICP-OES in the crude oil aqueous extract. Fuel.

[3] Lopez-Moreno C., Viera Perez I., Urbano A.M. (2016). Development and validation of an ionic chromatography method for the determination of nitrate, nitrite and chloride in meat. Food Chem..

[4] Robaina N.F., Feiteira F.N., Cassella A.R., Cassella R.J. (2016). Determination of chloride in brazilian crude oils by ion chromatography after extraction induced by emulsion breaking. J. Chromatogr. A.

[5] Diaz P., Gonzalez Z., Granda M., Menéndez R., Santamaría R., Blanco C. (2014). Evaluating capacitive deionization for water desalination by direct determination of chloride ions. Desalination.

[6] Rocha D.L., Rocha F.R.P. (2013). An environmentally friendly flow-based procedure with photo-induced oxidation for the spectrophotometric determination of chloride in urine and waters. Microchem. J..

[7] Bujes-Garrido J., Arcos-Martinez M.J. (2016). Disposable sensor for electrochemical determination of chloride ions. Talanta.

[8] Bujes-Garrido J., Arcos-Martinez M.J. (2017). Development of a wearable electrochemical sensor for voltammetric determination of chloride ions. Sens. Actuators B.

[9] De Graaf D.B., Abbas Y., Bomer J.G., Olthuis W., van den Berg A. (2015). Sensor–actuator system for dynamic chloride ion determination. Anal. Chim. Acta.

[10] Bonta M., Eitzenberger A., Burtscher S., Limbeck A. (2016). Quantification of chloride in concrete samples using LA-ICP-MS. Cem. Concr. Res..

[11] Kaur H., Singh J., Chopra S., Kaur N. (2016). Calix[4]arene based dipodal receptor nanohybrids for selective determination of chloride ions in aqueous media. Talanta.

[12] Gorbunova M.O., Bayan E.M., Voitsikhovskaya E.V. (2010). A glucotest for the quality control of food raw materials and products. J. Anal. Chem..

[13] Almeida M.I.G.S., Jayawardane B.M., Kolev S.D., McKelvie I.D. (2018). Developments of microfluidic paper-based analytical devices (μPADs) for water analysis: A review. Talanta.

[14] Morbioli G.G., Mazzu-Nascimento T., Stockton A.M., Carrilho E. (2017). Technical aspects and challenges of colorimetric detection with microfluidic paper-based analytical devices (μPADs)—A review. Anal. Chim. Acta.

[15] Cuartero M., Crespo G.A., Bakker E. (2015). Paper-Based Thin-Layer Coulometric Sensor for Halide Determination. Anal. Chem..

[16] Gorbunova M.O., Bayan E.M. (2017). A rapid field test method for the determination of hydrogen sulfide and sulfides in waters with gas preextraction. J. Anal. Chem..

[17] Pla-Tolós J., Moliner-Martínez Y., Verdú-Andrés J., Casanova-Chafer J., Molins-Legua C., Campíns-Falcó P. (2016). New optical paper sensor for in situ measurement of hydrogen sulphide in waters and atmosphere. Talanta.

[18] Gorbunova M.O., Zhikhareva I.N. (2003). A Test Method for Determining Active Chlorine in Drinking Water. J. Anal. Chem..

[19] Gorbunova M.O., Bayan E.M., Shevchenko A.V., Kulyaginova M.S. (2017). Digital colorimetric determination of chlorides in water using gas extraction and methyl orange. Anal. I Kontrol.

[20] Danchana K., Maya F., Wilairat P., Uraisin K., Cerdà V. (2015). Spectrophotometric determination of bromide in water using the multisyringe flow injection analysis technique coupled to a gas-diffusion unit. Anal. Methods.

[21] Loh L.J., Bandara G.C., Weber G.L., Remcho V.T. (2015). Detection of water contamination from hydraulic fracturing wastewater: A μPAD for bromide analysis in natural waters. Analyst.

[22] Apyari V.V., Gorbunova M.O., Shevchenko A.V., Furletov A.A., Volkov P.A., Garshev A.V., Dmitrienko S.G., Zolotov Y.A. (2018). Towards highly selective detection using metal nanoparticles: A case of silver triangular nanoplates and chlorine. Talanta.

[23] Apyari V.V., Furletov A.A., Garshev A.V., Volkov P.A., Gorbunova M.O., Shevchenko A.V. (2017). Preparation of reagent indicator papers with silver triangular nanoplates for chemical analysis. Mosc. Univ. Chem. Bull..

[24] Gorbunova M.O., Shevchenko A.V., Apyari V.V., Furletov A.A., Volkov P.A., Garshev A.V., Dmitrienko S.G. (2018). Selective determination of chloride ions using silver triangular nanoplates and dynamic gas extraction. Sens. Actuators B.

[25] Priyadarshini E., Pradhan N. (2017). Gold nanoparticles as efficient sensors in colorimetric detection of toxic metal ions: A review. Sens. Actuators B.

[26] Fang C., Dharmarajan R., Megharaj M., Naidu R. (2017). Gold nanoparticle-based optical sensors for selected anionic contaminants. Trends Anal. Chem..

[27] Lim M.-C., Kim Y.-R. (2016). Analytical Applications of Nanomaterials in Monitoring Biological and Chemical Contaminants in Food. J. Microbiol. Biotechnol..

[28] Nie G., Li G., Wang L., Zhang X. (2016). Nanocomposites of polymer brush and inorganic nanoparticles: Preparation, characterization and application. Polym. Chem..

[29] Abalde-Cela S., Carregal-Romero S., Paulo Coelho J., Guerrero-Martínez A. (2016). Recent progress on colloidal metal nanoparticles as signal enhancers in nanosensing. Adv. Colloid Interface Sci..

[30] Ahmad R., Griffete N., Lamouri A., Felidj N., Chehimi M.M., Mangeney C. (2015). Nanocomposites of Gold Nanoparticles@Molecularly Imprinted Polymers: Chemistry, Processing, and Applications in Sensors. Chem. Mater..

[31] Choi I. (2016). Recent Advances in Nanoplasmonic Sensors for Environmental Detection and Monitoring. J. Nanosci. Nanotechnol..

[32] Yue G., Su S., Li N., Shuai M., Lai X., Astruc D., Zhao P. (2016). Gold nanoparticles as sensors in the colorimetric and fluorescence detection of chemical warfare agents. Coord. Chem. Rev..

[33] Sharma R., Ragavan K.V., Thakur M.S., Raghavarao K.S.M.S. (2015). Recent advances in nanoparticle based aptasensors for food contaminants. Biosens. Bioelectron..

[34] Huynh K.A., Chen K.L. (2011). Aggregation Kinetics of Citrate and Polyvinylpyrrolidone Coated Silver Nanoparticles in Monovalent and Divalent Electrolyte Solutions. Environ. Sci. Technol..

[35] Godymchuk A., Karepina E., Yunda E., Bozhko I., Lyamina G., Kuznetsov D., Gusev A., Kosova N. (2015). Aggregation of manufactured nanoparticles in aqueous solutions of mono- and bivalent electrolytes. J. Nanopart. Res..

[36] El Badawy A.M., Luxton T.P., Silva R.G., Scheckel K.G., Suidan M.T., Tolaymat T.M. (2010). Impact of Environmental Conditions (pH, Ionic Strength, and Electrolyte Type) on the Surface Charge and Aggregation of Silver Nanoparticles Suspensions. Environ. Sci. Technol..

[37] Aryal S., Bahadur R., Bhattarai K.C.N., Kim C.K., Kim H.Y. (2006). Study of electrolyte induced aggregation of gold nanoparticles capped by amino acids. J. Colloid Interface Sci..

[38] Dzhardimalieva G.I., Uflyand I.E., Zhinzhilo V.A. (2022). Metal-polymer nanocomposites based on metal-containing monomers. Russ. Chem. Bull..

[39] Dzhardimalieva G.I., Uflyand I.E. (2020). Conjugated Thermolysis of Metal-Containing Monomers: Toward Core–Shell Nanostructured Advanced Materials. J. Inorg. Organomet. Polym. Mater..

[40] Mohamed M.A., Mansour S.A.A., Hussien G.A.M. (1994). Non-isothermal decomposition of silver maleate dihydrate and anhydrous silver fumarate. J. Therm. Anal..

[41] Smith G., Sagatys D.S., Dahlgren C., Lynch D.E., Bott R.C., Byriel K.A., Kennard C.H.L. (1995). Structures of the silver(I) complexes with maleic and fumaric acids: Silver(I) hydrogen maleate, silver(I) maleate and silver(I) fumarate. Z. Krist. New Cryst. Struct..

[42] Yudanova L.I., Logvinenko V.A., Yudanov N.F., Rudina N.A., Ishchenko A.V., Semyannikov P.P., Sheludyakova L.A., Alferova N.I., Romanenko A.I., Anikeeva O.B. (2013). Preparation of metal-polymer composites through the thermolysis of Fe(II), Co(II), and Ni(II) maleates. Inorg. Mater..

[43] Kolesnikova T.S., Zarubina A.O., Gorbunova M.O., Zhinzhilo V.A., Dzhardimalieva G.I., Uflyand I.E. (2022). Silver Itaconate as Single-Source Precursor of Nanocomposites for the Analysis of Chloride Ions. Materials.

[44] Gorbunova M.O., Apyari V.V., Baulina A.A., Garshina M.S., Kulyaginova M.S., Shevchenko A.V., Furletov A.A., Dmitrienko S.G., Zolotov Y.A. (2020). An improved step-by-step airflow/paper-based colorimetric method for highly selective determination of halides in complex matrices. Talanta.

[45] Gorbunova M.O., Garshina M.S., Kulyaginova M.S., Apyari V.V., Furletov A.A., Garshev A.V., Dmitrienko S.G., Zolotov Y.A. (2020). A dynamic gas extraction-assisted paper-based method for colorimetric determination of bromides. Anal. Methods.

[46] Gorbunova M.O., Baulina A.A., Kulyaginova M.S., Apyari V.V., Furletov A.A., Garshev A.V., Dmitrienko S.G. (2019). Determination of iodide based on dynamic gas extraction and colorimetric detection by paper modified with silver triangular nanoplates. Microchem. J..

[47] Gorbunova M.O., Bayan E.M. (2019). A novel paper-based sensor for determination of halogens and halides by dynamic gas extraction. Talanta.

[48] Gorbunova M.O., Baulina A.A., Kulyaginova M.S., Apyari V.V., Furletov A.A., Volkov P.A., Bochenkov V.E., Starukhin A.S., Dmitrienko S.G. (2019). Dynamic gas extraction of iodine in combination with a silver triangular nanoplate-modified paper strip for colorimetric determination of iodine and of iodine-interacting compounds. Microchim. Acta.

[49] Uflyand I.E., Gorbunova M.O., Zhinzhilo V.A., Kolesnikova T.S., Zarubina A.O., Baimuratova R.K., Dzhardimalieva G.I. (2022). Preparation of Ag/C Nanocomposites Based on Silver Maleate and Their Use for the Analysis of Iodine Ions. J. Compos. Sci..

[50] Apyari V.V., Dmitrienko S.G. (2008). Using a digital camera and computer data processing for the determination of organic substances with diazotized polyurethane foams. J. Anal. Chem..

[51] Skoog D.A., West D.M., Holler F.J., Crouch S.R. (2014). Fundamentals of Analytical Chemistry.

